# Hyaluronan and Collagen in Human Hypertrophic Cardiomyopathy: A Morphological Analysis

**DOI:** 10.1155/2012/545219

**Published:** 2012-07-26

**Authors:** Martin Hellström, Anna Engström-Laurent, Stellan Mörner, Bengt Johansson

**Affiliations:** ^1^Department of Public Health and Clinical Medicine, Medicine, Umeå University, 90187 Umeå, Sweden; ^2^Department of Clinical Science, Section for ENT and Department of Radiation Sciences, Oncology, Umeå University, SE-90187 Umeå, Sweden

## Abstract

*Introduction*. The hypertrophic cardiomyopathy (HCM) disease process is not only limited to cardiomyocyte abnormalities but also engages the extracellular matrix. Hyaluronan (HA) and its receptor CD44 are involved in cellular growth and tissue proliferation but have so far been less studied in myocardial hypertrophy. In HCM, collagens are abundant but their histological distribution and relation to hyaluronan have not been described. *Material and Methods*. Myocardial specimens from 5 patients with symptomatic left ventricular tract obstruction undergoing myectomy due to HCM were processed for histochemistry and immunohistochemistry. *Results*. HA staining was more intense in HCM patients. The histological distribution of HA was the same in patients and controls, that is, interstitial staining including the space between cardiomyocytes, in fibrous septa, and in the adventitia of intramyocardial blood vessels. CD44 was not detected in the myocardium of patients or controls. Collagen I showed the same general localisation as HA but detailed distribution differed. *Conclusions*. This is the first study that describes the distribution of hyaluronan in human HCM. HA staining is more intense in HCM patients but without coexpression of its receptor CD44, at least not in the chronic phase of HCM. HA and collagen I have the same localisation.

## 1. Introduction

Hypertrophic cardiomyopathy (HCM) is characterised by left and/or right ventricular hypertrophy, with predominant involvement of the interventricular septum [1]. There are many genetic and clinical variants of HCM, with a broad spectrum of clinical manifestations, ranging from asymptomatic individuals to severe symptoms and early death [2]. Furthermore, HCM may cause ventricular arrhythmias and is an important cause of sudden cardiac death in children, young adults, and athletes.

The myocardial hypertrophy is the key pathophysiological feature and is characterised by abnormal cardiomyocytes, disarray, and interstitial fibrosis, thus involving the extracellular matrix (ECM). The main components of the ECM are collagens and glycosaminoglycans (GAGs). In the hypertrophic heart, the best-characterised ECM components are the collagens I and III [3]. Collagens are excellent at withstanding tensile forces and it has been discussed how changes in cardiac hypertrophy can affect function, for example, increased stiffness [4, 5] leading to diastolic dysfunction. Collagen fibres are made up by three protein chains that form a stiff triple-stranded helical structure and are distributed throughout the entire myocardium [3]. In nonhuman primate myocardium, collagen I represents 85% and collagen III 11% of the total collagen protein content [6]. Comparing weight, collagen is the dominating ECM component, but by volume GAGs occupy most of the extracellular space. There are only few reports on the expression and distribution of the GAGs, of which hyaluronan (HA) is the main molecule [7, 8].

HA is an extracellular and cell surface associated polysaccharide composed of repeating disaccharides of glucuronic-acid and N-acetylglucosamine [9]. It differs from the other GAGs in that it is the only nonsulphated member and is produced at the inner side of the cell membrane by activation of any of the three hyaluronan synthesising enzymes [10]. Apart from structural properties, the molecule is viscoelastic and has barrier and sieve functions that regulate transport and migration of other molecules and cells through the tissues [9]. The polysaccharide also has cell biological functions such as coregulating cellular events during embryonic development, healing processes, and, inflammation and is involved in tumour development [11]. The main HA receptor is CD44. CD44 is a transmembrane glycoprotein and involved in cell-cell and cell-matrix interactions and signal transduction. The receptor exists on a variety of cells and in different isoforms.

We have previously reported on the distribution of HA and CD44 in the heart of newborn and adult rats [12] and showed that there is an increased synthesis of HA and CD44 in an experimental rat model of cardiac hypertrophy [13], but the relevance of these components in human HCM is unknown. In the present study, we have used histological methods to explore the distribution of HA, its receptor CD44, and collagen I in myectomies from patients with HCM.

## 2. Material and Methods

### 2.1. Patients and Tissues

Five individuals with HCM and significant left ventricular outflow tract obstruction, scheduled for surgical myectomy, were included in the study: three women and two men, aged 28 to 73 years (Table 1). Four of them had no family history of HCM and were considered as sporadic and one of them had familial HCM. All had symptoms consistent with the diagnosis of HCM and had significant left ventricular outflow tract obstruction at rest and/or at stress. Criterium for the diagnosis of HCM was left ventricular hypertrophy, with a diastolic wall thickness of 15 mm, without other obvious reason for hypertrophy such as arterial hypertension or valvular heart disease. Informed consent was obtained from each patient. Samples from left ventricular myocardium were obtained at surgical septal myectomy. Control myocardium from the left ventricle was collected at autopsy of five previously healthy individuals that had died a sudden unnatural death. All myocardial specimens were mounted in OTC compound (Tissue Tek, Sakura Finetek, Zoeterwoude, Netherlands) and frozen in liquid propane chilled with liquid nitrogen. Until further processing, the tissues were stored at −80°C. The protocol was approved by the Ethics Committee of the Medical Faculty, Umeå University. The autopsy specimens were collected in agreement with Swedish laws and regulations on autopsy and transplantation.

Three different probes/antibodies were used, respectively, for localisation of HA, CD44, and collagen I. The frozen cardiac tissues were sectioned in 10 *μ*m sections using a cryostat (Leica CM3000).

### 2.2. Histochemical Staining for HA

For the localisation of HA, a hyaluronan binding protein (HABP), kindly donated from Corgenix Inc. (Westminster, CO, USA), was used. The isolation and biotin labelling of the HABP have been described before [14].

The slides were thawed for 5 min and washed in a fixation solution (2% formalin and 0.5% glutaraldehyde) in 0.1 M phosphate-buffered saline PBS, pH 7.35, for 2.5 min and then washed in PBS and incubated with a solution of 3% H_2_O_2_ in methanol for 5 min, washed once in distilled water, once in PBS, and incubated with bovine serum albumin (10 mg/mL) for 30 min to block nonspecific binding sites. All slides were then washed in PBS. Control slides were preincubated with *Streptomyces* hyaluronidase 50 units/mL (Sigma, St. Louis, MO, USA), a selective carbohydrate-digesting enzyme, for 4 h at 37°C. This enzyme specifically degrades HA and therefore serves as a control, showing the specificity of the method. The slides were washed and incubated with HABP (1 : 40 dilution) at 4°C overnight. After washing in PBS, all slides were incubated with Vectastain-Elite Avidin-Biotin complex reagent (Vector Laboratories, Burlinggame, CA, USA) for 40 min, washed in PBS, and incubated for 5 min in a solution of 3′-diaminobenzidine (DAB). Following washing, Mayer's hematoxylin was used as counter staining. Finally, the sections were washed again, dehydrated, and cover slipped.

### 2.3. Immunohistochemical Staining for CD44

For the localisation of CD44, a purified mouse anti-human monoclonal antibody (Sigma, St. Louis, MO, USA) was used. After being thawed, the sections were incubated in a solution of 2% formalin and 0.5% glutaraldehyde in 0.1M Tris-buffered saline (TBS), pH 7.35, for 2.5 min, washed in TBS and then in citric acid buffer for 2 × 6 min in a microwave oven at a setting of 98°C at 750 W. After cooling, the slides were washed in distilled water, incubated with a solution of 0.3% H_2_O_2_ in methanol for 30 min, rinsed in distilled water, and then washed in 0.05 M TBS pH 7.4. After being incubated with normal rabbit serum (DAKO A/S, Copenhagen, Denmark) (dilution to 1 : 20) for 30 min, the experimental slides were incubated with the mouse anti-human CD44 antibody at 1 : 5 dilution and the control slides with normal serum for 30 min. After being washed in TBS, all slides were incubated with a biotinylated rabbit anti-mouse antibody (DAKO A/S), at 1 : 300 dilution for 30 min. Following a wash, the sections were incubated with a Vectastain-Elite Avidin-Biotin complex reagent (Vector Laboratories, Burlinggame, CA, USA) for 40 min, washed again for 3 × 10 min in TBS, and incubated 5 min in a solution of DAB (Vector Laboratories). After being washed in tap water, the sections were finally dehydrated and cover slipped.

### 2.4. Immunohistochemical Staining for Collagen I

After 5 min thawing, the slides were incubated for 5 min in 3% H_2_O_2_, washed in distilled water, and incubated in PBS (0.01 M; pH 7.4) for 5 min. All slides were incubated with normal swine serum (DAKO A/S, Copenhagen, Denmark) (dilution 1 : 20) and experimental slides where then incubated with rabbit-anti-human collagen I antibody, dilution 1 : 1200, for 1 hour. Both control and experimental slides were then washed twice in PBS, incubated with a biotinylated swine-anti-rabbit antibody (DAKO A/S, Copenhagen, Denmark) (dilution 1 : 300) for 30 min, and washed trice in PBS. After incubation with a Vectastain-Elite Avidin-Biotin complex reagent for 40 min, the sections were washed for 3 × 10 min in PBS and incubated for 5 min in a solution of DAB. Following a wash in tap water for 5 min, the sections were dehydrated and cover slipped. Due to limited access to normal heart tissue, only HCM samples were available for collagen I staining. 

The HABP-stained sections were evaluated by three of the authors (Martin Hellström, Bengt Johansson and Anna Engström-Laurent). The staining intensity was recorded using a three-graded scale: + = weak, ++ = moderate, and +++ = intense. Staining intensity of collagen I stained sections was not graded, as no control specimens were available. Only localization in HCM specimens is described.

## 3. Results

### 3.1. General Histology

**Figure 1:**
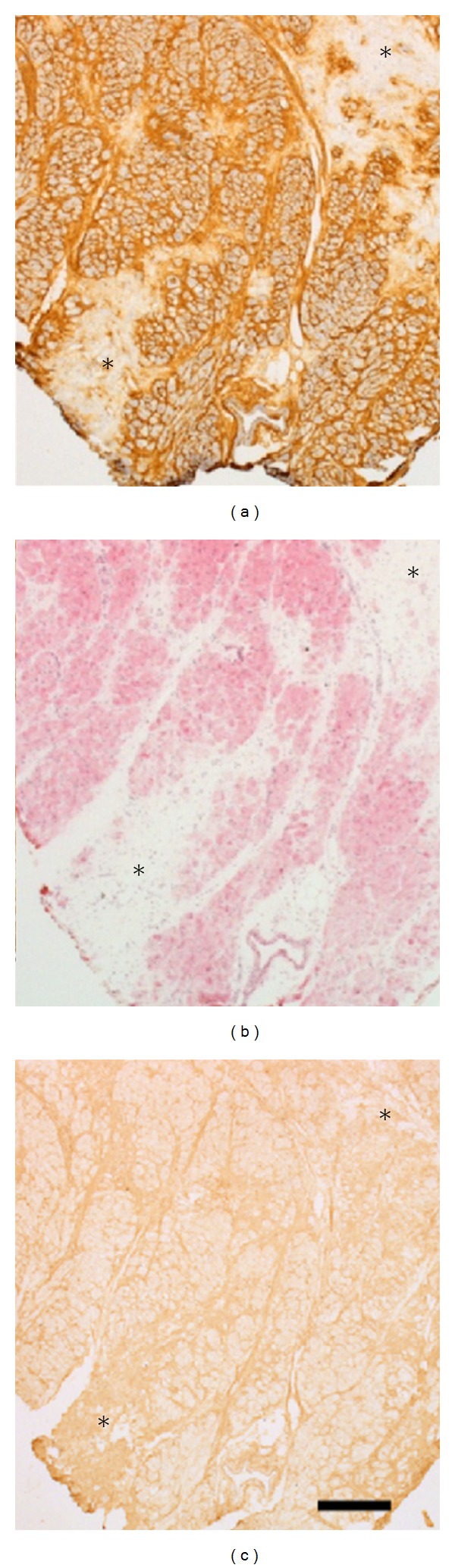
Serial sections from patients with HCM stained with HA-binding probe (a), hematoxylin-eosin (b), and for visualization of collagen I (c). The fibrotic “scars” (marked*) show weak or no HA staining (a) but abundant collagen I staining (c). The bar in C represents 500 *μ*m.

**Figure 2:**
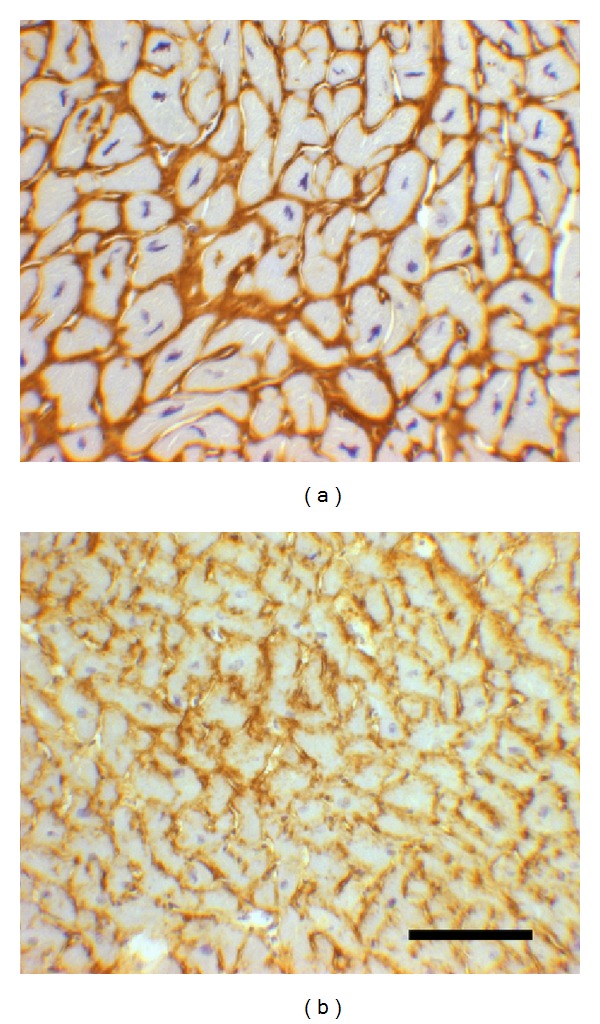
Sections of myocardium from patients with HCM (a) and control (b) stained for visualisation of HA. In HCM, there is marked cardiomyocyte hypertrophy and variation in cell size (a) compared with controls (b). The bar in B represents 100 *μ*m.

The HCM specimens (*n* = 5) showed variations in cardiomyocyte size and form but the majority of the cardiomyocytes were hypertrophied. HCM specimens showed varying degrees of fibrosis with increased intercellular fibrosis, perivascular fibrosis, thickened fibrous septa, and patches of focal fibrosis. All findings were in line with the expected histological findings in HCM. The control specimens (*n* = 5) showed normal morphology (Figures 1(a), 1(b), 1(c), 2(a) and 2(b)).

### 3.2. HA

The HCM samples showed more intense HA staining compared to controls although the distribution was similar (Table 2). HA was detected in the intercellular space, in fibrous septa, around intramyocardial blood vessels, and in HCM myocardium in patches of focal fibrosis (Figure 2(a)). Some of these focal scars were unstained although they appeared as fibrotic in routine histological stainings (Figure 1(a)).

### 3.3. CD44

There was no CD44 staining in HCM or in control specimens.

### 3.4. Collagen I

In HCM, collagen I was located between cardiomyocytes, in fibrous septa, in the perivascular space, and in focal scars (Figure 1(c)). Only HCM samples were available for collagen I staining due to limited access to normal heart tissue.

## 4. Discussion

This study is the first to report on the distribution of the ECM molecules HA and CD44 based on analyses on myectomy specimens from clinically well-characterised HCM patients with symptomatic left ventricular outflow tract obstruction. The morphological appearance of the HCM specimens in this study displayed widened intercellular space, hypertrophic cardiomyocytes, and an increase in both volume and numbers of fibrous septa. The significant hypertrophy of the cardiomyocytes in this material studied has been described earlier [15]. There was no difference in the localisation of HA and collagen I in the HCM specimens. That HA is present and distributed throughout the myocardium in both healthy and pathological conditions has also been described by us earlier in a hypertrophic rat model [13]. However, the intensity of the HA-staining of the HCM specimens was clearly increased compared to normal heart tissue. All manifestations of fibrosis such as perivascular, interstitial, and focal showed a distinct staining intensity of both HA and collagen I. In some of the HCM specimens, a few focal fibrosis-like scars were lacking HA staining, the reason for which can only be speculated on. The collagen I staining did not show this pattern and one explanation may be that the deposited collagen bundles are too dense to allow space for HA.

Considering the structural and cell biological properties of HA, the presence of the molecule in both normal and pathological myocardium is an interesting observation. In the normal heart HA provides, among other functions, both stability and protection. The structural and cell biological effects of HA in HCM are unknown, but most probably both cell signalling and cell catabolism are involved. In HCM, it can be discussed whether the increase in HA is an adaptive response to the contractile dysfunction and/or a part of the pathological process causing cardiomyocyte hypertrophy and fibrosis. An HA rich coat around the cardiomyocytes in the normal heart may facilitate cell-to-cell contacts and facilitate the propagation of electrical impulses. In hypertrophy, the effects might be the reverse with a thickened HA coat that block essential cell-cell and cell-matrix communications. This may cause further hypertrophy, thus, creating a vicious circle. One proposed theory is that the increased ECM of the heart may have additional negative effect on the contractile dysfunction besides the systolic dysfunction that is due to the sarcomeric genetic defect [4, 5]. Furthermore, the expanded ECM probably contributes to diastolic dysfunction that is a feature of myocardial hypertrophy with stiff ventricular walls.

It seems that normal/healthy hearts do not express CD44. This is consistent with earlier experimental data [12, 13] and data on human hearts [16]. In an experimental rat model of pressure-induced hypertrophy, CD44 was expressed in and around myocardial vessels in the early phase of the hypertrophic process [13]. In the present study, the HCM is in a chronic stage which might be an explanation of the absence of CD44. However, conflicting data have been reported with expression of CD44 both in the endocardium of patients with congestive and ischemic heart failure and in donor hearts [17]. In our study cardiac decompensation with clinical heart failure was not present which also may explain the absence of CD44 staining. Nevertheless, it seems likely that HA can reside in the myocardium without colocalisation with CD44.

A limitation of our study is the restricted availability of control biopsies. This makes statistical analyses difficult to perform and also limits further comparison. However, in spite of the semiquantitative method used in this study, the difference in staining indicates a true increase of HA in HCM compared to normal hearts. Hyaluronan was present in the interstitium including the space between cardiomyocytes and in fibrous septa and the same staining pattern was seen for collagen I. This is the first study describing the distribution of HA in human HCM and the data suggests that HA is involved in the remodelling process of HCM.

## Figures and Tables

**Table 1:** Clinical and echocardiography characteristics of patients with hypertrophic cardiomyopathy.

				Echocardiography					
Subject	Gender	Age (yrs)	Age at diagnosis (yrs)	IVSD	LVPWD	IVSD/LVPWD	LVEDD	LVESD	LVOT obstruction^∗^
				(mm)	(mm)		(mm)	(mm)	
1	F	45	43	20	20	1.0	38	12	Yes
2	F	28	27	21	14	1.5	43	18	Yes
3	F	73	66	19	12	1.6	40	21	Yes
4	M	43	41	19	10	1.9	39	24	Yes
5	M	68	66	20	11	1.8	46	30	Yes

IVSD: interventricular septum dimension in end diastole, LVPWD: left ventricular posterior wall dimension in end diastole, LVEDD: left ventricular end-diastolic diameter, LVESD: left ventricular end-systolic diameter, LVOT: left ventricular outflow tract, and *>30 mmHg at rest or >50 mmHg under stress.

**Table 2:** The HABP-stained sections were evaluated and the staining intensity was recorded using a three-graded scale: +: weak, ++: moderate, and +++: intense.

Tissue	Prep	Hyaluronan
HCM	1	++
	2	+++
	3	+++
	4	+++
	5	++

Normal	1	++
	2	+
	3	++
	4	+++
	5	+

## Abbreviations

HCM: Hypertrophic cardiomyopathy
HA: Hyaluronan
ECM: Extracellular matrix
GAG: Glycosaminoglycan
HABP: Hyaluronan binding protein
PBS: Phosphate-buffered saline
DAB: 3, 3′-diaminobenzidine
TBS: Tris-buffered saline.
